# Referral Systems to Integrate Health and Economic Strengthening Services for People with HIV: A Qualitative Assessment in Malawi

**DOI:** 10.9745/GHSP-D-16-00195

**Published:** 2016-12-23

**Authors:** Clinton Sears, Zach Andersson, Meredith Cann

**Affiliations:** a FHI 360, Washington, DC, USA.

## Abstract

**Background:**

Supporting the diverse needs of people living with HIV (PLHIV) can help reduce the individual and structural barriers they face in adhering to antiretroviral treatment (ART). The Livelihoods and Food Security Technical Assistance II (LIFT) project sought to improve adherence in Malawi by establishing 2 referral systems linking community-based economic strengthening and livelihoods services to clinical health facilities. One referral system in Balaka district, started in October 2013, connected clients to more than 20 types of services while the other simplified approach in Kasungu and Lilongwe districts, started in July 2014, connected PLHIV attending HIV and nutrition support facilities directly to community savings groups.

**Methods:**

From June to July 2015, LIFT visited referral sites in Balaka, Kasungu, and Lilongwe districts to collect qualitative data on referral utility, the perceived association of referrals with client and household health and vulnerability, and the added value of the referral system as perceived by network member providers. We interviewed a random sample of 152 adult clients (60 from Balaka, 57 from Kasungu, and 35 from Lilongwe) who had completed their referral. We also conducted 2 focus group discussions per district with network providers.

**Findings:** Clients in all 3 districts indicated their ability to save money had improved after receiving a referral, although the percentage was higher among clients in the simplified Kasungu and Lilongwe model than the more complex Balaka model (85.6% vs. 56.0%, respectively). Nearly 70% of all clients interviewed had HIV infection; 72.7% of PLHIV in Balaka and 95.7% of PLHIV in Kasungu and Lilongwe credited referrals for helping them stay on their ART. After the referral, 76.0% of clients in Balaka and 92.3% of clients in Kasungu and Lilongwe indicated they would be willing to spend their savings on health costs. The more diverse referral network and use of an mHealth app to manage data in Balaka hindered provider uptake of the system, while the simpler system in Kasungu and Lilongwe, which included only 2 referral options and use of a paper-based referral tool, seemed simpler for the providers to manage.

**Conclusions:**

Participation in the referral systems was perceived positively by clients and providers in both models, but more so in Kasungu and Lilongwe where the referral process was simpler. Future referral networks should consider limiting the number of service options included in the network and simplify referral tools to the extent possible to facilitate uptake among network providers.

## Abstract (precis)

Two types of referral systems were implemented in this low-resource context: (1) a simple paper-based system connecting clinical HIV and nutrition support to village savings and loans services, and (2) a complex mHealth-based system with more than 20 types of health, economic strengthening, livelihoods, and food security services. Clients reported the referrals improved their health and nutrition and ability to save money in both models but more with the simple model. Providers had difficulty using the mobile app under the mHealth system, even after repeated trainings, considerable ongoing technical assistance, and multiple rounds of revisions to the interface.

## INTRODUCTION

Every week around the world, more than 3,600 children and 25,000 adults die from HIV.^1^ The majority come from resource-poor and poverty-stricken populations who, on a daily basis, face challenges to accessing and using health services. The U.S. President's Emergency Plan for AIDS Relief (PEPFAR) outlines an ambitious agenda to achieve the Joint United Nations Programme on HIV/AIDS (UNAIDS) 90-90-90 goals: by 2020, 90% of people living with HIV (PLHIV) will be diagnosed, 90% of those diagnosed will be on antiretroviral therapy (ART), and 90% of those on ART will be virally suppressed.^1^

People living with HIV face both individual and structural factors that make reaching the 90-90-90 goals a challenge. Poverty, in particular, can exacerbate the challenges that PLHIV face when prioritizing scarce resources such as time, money, and energy.^2^ In Malawi, for example, the cost of ART is subsidized by the government, but there may be other costs involved in client care depending on the facility and whether it is public or private. Simply put, for impoverished PLHIV, the disease often forces a choice between accessing care and treatment or food.^3^ Poor health infrastructure, long wait times, distance between residences and clinical care, and decreased access to testing and care services worsen conditions and can contribute to a reshuffling of priorities in which ART adherence suffers.^2^ In a vicious cycle, the time, energy, and money often required for HIV care can directly strain such things as household food security and payment of school fees. These factors can lead to unhealthy acceptance of risk whereby clients either consciously decide they cannot make it to a clinic to collect their medicine or pick up their medicine only to discover that transport expenses or lost opportunity costs have made them unable to afford the food needed to avoid nausea and improve medicinal effectiveness.

People living with HIV face both individual and structural barriers that make reaching the UNAIDS 90-90-90 goals a challenge.

Formalizing relationships between clinical and relevant community stakeholders, including economic strengthening, livelihoods, and food security organizations, while building the capacity and buy-in of these stakeholders around the potential benefits of sharing client information with each other to more holistically address client needs, is a relatively new concept. However, research has shown that targeting the multiple but interrelated needs of PLHIV can produce more sustainable results.^3^^–^^5^ Limited evidence on the effect of community linkages demonstrates important associations between community support and improved clinical outcomes for PLHIV.^6^^–^^8^ Further, Okello et al. note that "… strengthening the capacity of community-based organizations to play a frontline role in implementing interventions in health, economic and social development is a prerequisite for transformational development."^4^ A randomized controlled trial conducted by Weiser et al. in 2012 demonstrated "… that a potentially sustainable agricultural and financial intervention improved immunologic and virologic outcomes, food security and diet quality for HIV-infected individuals."^5^ This supports previous research attesting the critical role of poverty and food security alleviation in improving health outcomes such as adherence to ART and retention in care.^9^ The psychological benefits derived from certain livelihood support opportunities, such as membership in a village savings and loan association (VSLA), should also not be discounted. As Yager and colleagues note, integrated HIV and livelihoods programs can help empower PLHIV and may indirectly bolster self-esteem, improve client standing in the community, and reduce stigma that can also influence client decisions to make healthy choices.^3^

Targeting the multiple but interrelated health and non-health needs of PLHIV can produce more sustainable results.

Several recent studies have examined how different types of economic incentives, such as cash payments, prize-based systems, and vouchers for goods, may affect ART adherence or virologic suppression. Bassett et al. reviewed four randomized controlled trials of conditional economic incentives and found that all showed significant increases in ART adherence (as high as 30%) compared with control groups.^10^ Unfortunately, some benefits faded as soon as 8 weeks after the incentive intervention stopped. Solomon et al. explained how modest nonmonetary voucher incentives as part of an intervention in Chennai, India, were associated with higher rates of linkage to care, ART initiation, and retention in care.^11^

Whereas these incentive schemes reflect positive behavior change in the short-term, the Livelihoods and Food Security Technical Assistance II (LIFT) project hypothesized that formalizing and institutionalizing mechanisms for ongoing collaboration and referral between service providers from disparate sectors can contribute to the well-being of PLHIV in the long-run. LIFT, a global technical assistance project with experience in 7 countries, connects existing service providers from multiple sectors (e.g., health, economic strengthening, agriculture, social protection) to one another. Collectively, LIFT, with support from the service providers, develops referral tools and standardizes a formal referral process that the providers use to track vulnerable clients through the referral system.

This article analyzes feedback from service providers and clients about how participation in integrated referral systems may contribute to improving client resilience and positive health outcomes. The LIFT team believes the assessment examined herein represents a unique venture—to the best of our knowledge, this has not been done before.

## PROGRAM DESCRIPTION

Led by FHI 360 with partners CARE International and World Vision International, the LIFT project provides technical assistance in multiple sub-Saharan African nations. Recognizing that HIV is complex, LIFT was designed to improve health outcomes for PLHIV by linking them to nonclinical economic strengthening, livelihoods, and food security services. While LIFT's focus is on improving the lives of PLHIV and orphans and vulnerable children, in practice the project's referral work is open to both PLHIV and people without HIV to ensure that referrals are not stigmatized as an HIV service. This also helps improve sustainability by making referrals broadly applicable to all service providers that make up a referral network.

LIFT maps existing economic strengthening, livelihoods, and food security service providers in the catchment area of health facilities and invites those providers to form referral networks while offering technical assistance to the nascent network. (LIFT does not work directly with individual Malawian clients.) The locally managed referral networks offer bidirectional clinic-to-community referrals to improve the health of individuals and the communities in which they live. The particular goal of clinic-to-community referral networks is to create a sense of shared responsibility and establish a platform for ongoing dialogue between the health system and the community to reduce factors that can be barriers to care, whether they are related to the clients or to the support system.^12^ Referral networks can include government, civil society, and community-based service providers, and they provide an important forum for information, education, and communication across these entities, many of which would not otherwise interact.

The LIFT project invites existing providers in a catchment area to form referral networks while offering technical assistance to the networks.

From October 2013 to April 2015, LIFT staff worked directly with a diverse group of local service providers from multiple sectors (public, private, government) and program areas (e.g., health, nutrition, agriculture, finance) in Balaka district of Malawi. LIFT staff carried out an extensive service enumeration and organizational network analysis to highlight the state of coordination and collaboration among the service providers at that time. LIFT led development of referral tools, trained service providers on appropriate use of these tools, and launched a cloud-based referral database using CommCare, an mHealth application, to manage client cases in real time.

In the Balaka referral network model, service providers administered a food security and vulnerability diagnostic tool to appropriately counsel clients as they decided which services included in the network directory would be the most beneficial. Clients were adult Malawians (ages 18 and above) who were registered for and offered a referral from one stakeholder in the referral network to another stakeholder. The providers read a consent script to these clients when they registered the clients, which noted that the referral network (including the LIFT project in a technical assistance role) might follow up with them in the future to assess the value of referrals. The providers made the referrals through the mHealth app, so that the receiving provider could open the app to monitor which clients to expect and easily confirm if the clients received the service. Use of the mHealth app also proved beneficial for LIFT to track and monitor referral data, since the data were uploaded to a cloud-based server in real time, rather than collected monthly.

In July 2014, LIFT began to design and implement a simplified referral system model. This second model connected health sector clients from Nutrition Counseling, Support, and Treatment (NCST) facilities in Kasungu and Lilongwe districts directly to community-based VSLAs. The NCST facilities participated in the Ministry of Health's NCST program to integrate nutrition and HIV services. LIFT capitalized on existing VSLAs in both districts by approaching them and successfully negotiating addition of the referral component to their portfolio. LIFT trained all referral stakeholders in Kasungu and Lilongwe to follow the NCST clinic-to-VSLA referral process and provided them with tools to help track clients to promote and verify referral completion. Whereas the Balaka referral network used a mobile phone-based data collection platform, the referral network in Kasungu and Lilongwe used a paper-based system. LIFT designed these two system models to achieve slightly different goals as summarized in Table 1.

**TABLE 1.** LIFT Referral Models in Malawi, by District

District	Referral Model Features	Referral Model Goals
**Balaka**	Linked clients to all community services that chose to be members of the referral network. LIFT conducted a thorough mapping of services and invited all interested organizations (government, CSO, NGO, etc.) to participate. Clients were expected to complete referrals themselves. Used CommCare, an mHealth app, for data collection and management. Providers made referrals for one service at a time to promote completion of the referral. There was no limit on the number of referrals a client could be given over time, although few (<1%) clients chose more than 1 referral. Full range of ES/L/FS services were included, based on what already existed in the community. LIFT did not create new services. Most popular services were microfinance, health, and government-supported services for agriculture and social welfare.	This first referral model was designed for local ownership and sustainability and featured a systems-level approach to referral network membership. This model also sought to accommodate clients across the vulnerability spectrum, offering referrals to existing economic strengthening services targeting less vulnerable households (such as microfinance), somewhat vulnerable households (such as savings groups or land rights education), and very vulnerable households (such as asset transfer).
**Kasungu and Lilongwe**	Linked clients directly from NCST sites to VSLA (clinic to community referral). When food aid was available at NCST sites, clients were also referred to food aid (within health facility referral). Clients were guided to VSLA by a referral volunteer to ensure completion. Used paper referral tools for data collection and management. Each client received one referral only. The options for referral were from the NCST site to VSLA, or vice versa, with referrals given to food aid on a limited basis. LIFT created VSLAs if none existed.	This second referral model was designed to be simpler to implement, in that it connected NCST clients directly to VSLA (and food aid, when available). In addition, this model took advantage of existing VSLAs to accelerate start-up time and reduce management costs.

Abbreviations: CSO, civil society organization; ES, economic strengthening; FS, food security; L, livelihood; LIFT, Livelihoods and Food Security Technical Assistance II project; NCST, Nutrition Counseling, Support, and Treatment; VSLA, village savings and loan association.

The referral network in Balaka district used an mHealth app while the systems in Kasungu and Lilongwe used a simple paper-based system.

Both referral models required ongoing technical assistance from LIFT for referral tool design and testing, data collection and management, and analysis. Although LIFT used the mHealth app for data management in Balaka, the service providers were slow to adapt to the smartphones and often preferred calling or sending an SMS to colleagues to check on client progress rather than using the tracking features of the app. This made it difficult to track clients and follow up with them in a timely manner in the event that they did not complete a referral. A further complication in Balaka was the sheer number of services available (more than 20), which had differing catchment areas, eligibility criteria, and funds available to serve clients. Despite the complications, referrals in Balaka were mostly given to microfinance, health, and government-supported agriculture and social welfare services.

In Kasungu and Lilongwe, the providers quickly adopted the simple paper referral forms. The only referral options were to/from an NCST site (which also sometimes disbursed food aid) or a VSLA. In addition, these sites had referral volunteers who physically led the client from one service to another. These differences in referral models resulted in improved client tracking through the referral volunteer in Kasungu and Lilongwe compared with Balaka and more manageable client flow by virtue of having only one service outside the NCST site. At the time of the assessment, LIFT had been supporting all sites for at least 1 year.

## METHODS

For this assessment, LIFT collected data from clients (adults ages 18 and above) who received a referral and from service providers who managed the referrals. We interviewed 152 clients (n = 60 from Balaka, n = 57 from Kasungu, and n = 35 Lilongwe) who completed referrals. LIFT randomly sampled these clients by selecting the nth client from a list of all 1,253 referral clients served at 9 NCST facilities across the 3 districts. In cases where at least 20 clients could not be identified from 1 facility, LIFT increased the sample size at another facility to meet the intended cumulative minimum sample of 100 clients.

We also conducted 2 focus group discussions (FGDs) with selected service providers from the referral network in each of the 3 districts, with group sizes ranging from 6 to 8 participants (Table 2). Certain stakeholders (such as Ministry of Health staff) were not local entities that participated in day-to-day referral activities and thus were not invited to participate in the FGDs. Non-health service providers were selected to participate in FGDs on the basis of demonstrated familiarity with and use of the referral tools. Health service providers were selected based on their commitment to regular use of referral tools, the extent of interaction with PLHIV clients, and their familiarity with clinic-to-community linkages. Selection en-sured a balance between NCST facility and community staff, as well as equal representation of both men and women. Familiarity with and consistent use of the referral tool was a selection factor because we wanted to receive constructive feedback from providers with actual experience engaging clients through the referral process.

**TABLE 2.** Focus Group Discussion Participants in Malawi, by District and Type of Service Provider

District	Health Care Providers	Non-Health Care Providers
**Balaka**	7 individuals representing 5 service providers (NCST facilities and community health organizations)	7 individuals representing 7 non-health service providers
**Kasungu**	8 individuals from 5 NCST facilities	9 individuals selected based on their role as Referral Volunteers (trained to accompany referral clients) and Village Agents (savings group leaders)
**Lilongwe**	8 individuals from 3 NCST facilities	8 individuals selected based on their role as Referral Volunteers (trained to accompany referral clients) and Village Agents (savings group leaders)

Abbreviation: NCST, Nutrition Counseling, Support, and Treatment.

### Data Collection Instruments

**Client perception data** were collected through structured interviews using a combination of Likert-scale, agree/disagree, and free-response questions. The intent was to collect data from clients (nearly 70% of whom were PLHIV) to help key stakeholders in Malawi improve current and future referral operations and to document the impact of referrals on client lives. The interviews sought to address how clients felt about referrals by asking questions such as, *"What was your experience with the referral?"*, *"Did you like it?"*, and *"Did you understand it?"* Interviews were generally structured to elicit client perceptions of the referral process, impacts on savings and health, and ease of participation in the referral system. The interviews also asked questions related to quality of life, relations with community members, and perceived stigma.

To help ensure confidentiality and better understand the client perspective on referral impacts, LIFT hired and trained 5 data collectors to conduct the interviews with referral clients in the 3 districts. In Kasungu and Lilongwe, CARE staff coordinated the interviews, which were conducted on the grounds of the health facilities. In Balaka, district health facility staff were given lists of referred clients and tasked with contacting clients and scheduling interviews. The interviews in Balaka took place at health facilities as well as on the premises of Sue Ryder Foundation and Chinansi Foundation—2 community-based service providers active in health programming: In all 3 districts, one-on-one interviews were held between data collectors and clients in private settings to encourage clients to freely share their experience.

Prior to the first interview, the LIFT team developed a series of interview questions, loaded them into an Open Data Kit (ODK) survey, and deployed the survey on mobile tablets for use by data collectors. Question types included multiple choice, free answer, and a recorded story summarizing the client's experience. The interviews were conducted in the local language of Chichewa; after each interview was completed, recorded client stories were uploaded from the tablets, translated to English, and transcribed by the data collectors.

**Service provider perception data** were collected through FGDs with health staff (from NCST sites) and non-health staff by a trained FGD facilitator and note taker. The FGD discussion guides varied based on whether the service providers worked in the health or non-health sector. For example, it was only relevant for health service providers to discuss impacts of referral system participation on clinical recordkeeping and data collection, as non-health service providers do not maintain these client records. LIFT requested that staff who actively managed referrals (i.e., interacted with clients, explained the purpose and functions of the network, made referrals, received referred clients, etc.) participate in the FGDs.

The FGDs sought to address stakeholder feelings and perceptions of the value of referrals, asking questions such as, *"Has demand for your services increased as a result of participation in the referral network?"*, *"Do clients know about the referral network and understand what it is?"*, and *"Do clients ask for referrals?"* FGD facilitators were instructed to probe for data on the following domains as well: (1) value of membership in the referral network for health service providers vs. other service providers; (2) major constraints faced by clients in attending clinical ART appointments; (3) role of VSLA meetings in fostering adherence to and retention in HIV care and treatment (for health FGDs only); (4) the role of VSLA meetings in addressing issues related to stigma and psychosocial support for PLHIV; and (5) social funds and use of savings for health expenses.

### Ethical Approval and Training

The following key counterparts contributed to the initial design of referral systems and were informed about the purpose of this study: the Department of Nutrition, HIV and AIDS within the Ministry of Health (responsible for NCST sites); the District Councils of Balaka, Lilongwe, and Kasungu; and referral network service providers (with whom LIFT had a long-established relationship). The study team received ethical approval from Malawi's National Commission on Science and Technology (NCST) on June 17, 2015, and from FHI 360's Office of International Research Ethics (OIRE) on June 22, 2015. LIFT hired a team of 5 Malawian data collectors and trained them on proper research ethics, project background in Malawi, and assessment tools from June 22–26, 2015. Data collectors tested client interview questions and the FGD guide among themselves, allowing for refinement and accurate Chichewa language translation. Fieldwork began on June 29, 2015, and ended on July 17, 2015. Client interviews and FGDs were conducted by the data collection team in Chichewa. Interview responses were collected using Open Data Kit (ODK) on mobile tablets, and FGD audio was recorded after receiving the consent of the participants. LIFT's partner in Malawi, CARE, managed English translation and transcription of FGD audio from Lilongwe after receiving recordings from the hired facilitator.

### Fieldwork

Fieldwork took place from June 22 to July 17, 2015. All tools were implemented concurrently to maximize efficiency of staff time. Table 3 provides a schedule of where and when each tool was implemented.

**TABLE 3.** LIFT Fieldwork Calendar in Malawi, June–July 2015

Activity	Jun 22–26, 2015	Jun 29–Jul 3, 2015	Jul 6–10, 2015	Jul 13–17, 2015
Training	Held training for LIFT data collectors and FGD facilitators in Lilongwe			
Interviews with referral clients	Translated interview tool and instructions into Chichewa	Interviews with Kasungu clients	Interviews with Balaka clients	Interviews with Lilongwe clients
Focus group discussions with service providers	Translated FGD tool and instructions into Chichewa	FGDs with health and non-health providers in Kasungu and Lilongwe districts	FGDs with health and non-health providers in Balaka Began transcription and translation of FGD transcripts	Continued transcription and translation of FGD transcripts until completed by August 7

Abbreviations: FGD, focus group discussion; LIFT, Livelihoods and Food Security Technical Assistance II project.

### Data Management

LIFT staff in Washington, DC, managed analysis of English FGD transcripts and coded responses according to 7 general themes using Dedoose software: positive user experience; negative user experience; health (positive and negative); demand for and access to services; general awareness and comprehension of value; regular and appropriate use of referral tools following referral process; social impacts. Client interview data were compiled using ODK Aggregate and exported to Microsoft Excel for analysis by LIFT staff in Washington, DC.

## FINDINGS

### Client Perceptions

The following sub-sections present clients' perceptions of the benefits of the referral system organized by subject matter: health benefits, savings benefits, household benefits, and perceptions of the referral process (Table 4).

**TABLE 4.** Percentage of Referral Clients in Malawi Confirming Referral Benefits, by District, 2015

Referral Benefit	Balaka	Kasungu and Lilongwe
**Health Benefits**		
Feel they are better able to stay on medication as result of referral	72.7%	95.7%
Willing to spend savings on health costs after referral	76.0%	92.3%
Attribute improvement in health to service received via referral	60.9%	81.1%
Attribute improvement in nutrition to service received via referral	52.2%	70.8%
**Savings Benefits**		
Able to save more money after referral	56.0%	85.6%
**Household Benefits**		
Had household savings before referral	63.3%	41.6%
Had household savings after referral	66.7%	81.4%
**Referral Process and Service Access**		
Knew of economic strengthening service availability before referral	65.0%	44.2%
Found referral process user-friendly	60.9%	81.1%
Reported they will continue using service after referral	68.3%	96.7%

#### Health Benefits

Client interview data suggest that the referral approaches used in Balaka and in Kasungu and Lilongwe were associated with different health and livelihoods effects. Nearly 70% of all clients interviewed had HIV infection. Many PLHIV attributed an improvement in their health to the services they received as a result of participating in the referral system. In Balaka, 72.7% of clients thanked referrals for helping them stay on their medicine. In Kasungu and Lilongwe this positive attribution soared to 95.7%. Equally important, 76.0% of clients in Balaka and 92.3% of clients in Kasungu and Lilongwe indicated they would be willing to spend their savings on health costs. When asked about improvement in health and nutrition that could be attributed to the referral(s), 60.9% of Balaka respondents reported improved health and 52.2% reported improved nutrition, while Kasungu and Lilongwe fared even better with 81.1% of respondents reporting improved health and 70.8% reporting improved nutrition.

About 73% of PLHIV in Balaka and 96% in Kasungu and Lilongwe attributed their participation in the referral network to helping them stay on their medicine.

Clients shared that referrals helped them better understand reasons to stay on their ART medications and cited positive results such as boosted immunity, less frequent illnesses, gained strength, and reduced viruses, among others. One client summarized the impact of his referral to a VSLA, saying:

*I was sick for a very long time, and I did not have money. Since I joined the program, I am now able to borrow money to go to the hospital. I recommend that this program should continue because when I am in need, I am able to go and borrow money.* (60–65-year old man with HIV infection; Kasungu District Hospital, Kasungu)

Another client explained how the economic service he received increased his ability to take food with his medications:

*… [The project] has helped a lot of people to be linked to groups where they can find money to buy food that is scarce around the house. … the money we borrow from the [VSLA] allows us to buy the food we need so that the medicine is effective in our bodies.* (45–50-year old man with HIV infection; Nathenje Health Centre, Lilongwe)

#### Savings Benefits

In Kasungu and Lilongwe, 85.6% of clients were able to save more money after they received their referral. One client explained the direct effect of her referral to a VSLA:

*When I was linked to the CARE group, things improved in my house especially that now [I] am able to go to the group and borrow money for school fees and also buy household items.* (40–45-year old woman with HIV infection; Kasungu District Hospital, Kasungu)

In Balaka, 56.0% of clients reported being able to save more money after their referral. It is important to reiterate that the service options available to referral clients through the Balaka referral approach were more diverse and included linkages to VSLA as well as other types of support, such as food and agriculture or education.

56% of referral clients in Balaka and about 86% in Kasungu and Lilongwe reported being able to save money after their referral.

#### Household Benefits

In Balaka, 30.0% of clients reported having a family member who had HIV infection, and 8.3% reported that they or someone else in their family had been clinically assessed as malnourished at some point during the year prior to the interview. In Kasungu and Lilongwe, the household burden of HIV and malnutrition was much higher: 59.8% of clients reported another PLHIV in their household, and 46.7% reported at least one case of malnourishment in the household.

Before the referral, only 63.3% of clients in Balaka and 41.6% of clients in Kasungu and Lilongwe reported having household savings. After the referral, 66.7% of clients in Balaka and 81.4% in Kasungu and Lilongwe reported an improved household situation.

One client explained the new economic opportunities afforded to his family through a VSLA:

*I belong to a VSL group and I have seen benefits from there. I am now weaving baskets and selling them. My wife is also doing business of brewing beer after giving her capital from the money I borrowed from the VSL group. Now my household has improved in terms of food security.* (40–45-year old man with HIV infection; Diamphwe Health Centre, Lilongwe)

Another client explained how an entire family could benefit from the money saved and borrowed:

*When I joined the referral process of LIFT II, I was connected to the VSL group and now I am able to borrow money from the group and use it for my household's welfare, such that my household and I have improved. My family and I were undernourished.* (35–40-year old woman with HIV infection; Nathenje Health Center, Lilongwe)

#### Referral Process

At the time of referral, only 65.0% of clients in Balaka and 44.2% of clients in Kasungu and Lilongwe reported knowing about an economic strengthening service available nearby. Clients generally found the referral process linking clinical care to community support to be easy to understand and not overly burdensome. In Balaka, 60.9% of clients found the process user-friendly, and 81.1% of clients in Kasungu and Lilongwe agreed. In Balaka, 68.3% of respondents said they intend to continue to use the services for which they were referred, suggesting high satisfaction and value. In Kasungu and Lilongwe, this rose to 96.7% of clients.

About 61% of referral clients in Balaka and 81% in Kasungu and Lilongwe found the referral process user-friendly.

### Service Provider Perceptions

FGDs explored service provider roles as members of a referral network, particularly regarding the ease of operations and process adoption, level of effort required to carry out referral responsibilities, successes and failures witnessed or experienced, and the perceived utility of the system for their clients. In general, stakeholders felt the referral system connected clients to new services and they believed the system was positively affecting their clients' lives.

#### Provider Perceptions of Health Benefits to Clients

The majority of health care providers participating in the FGDs attributed client participation in the referral system with improved client health. One provider from Diamphwe Health Centre in Lilongwe noted:

It has benefited people who are HIV positive to be open and they should not be afraid if they want to go to the hospital.

Improvements in social cohesion and psychosocial support were also cited, with a non-health care provider responsible for overseeing several VSLAs in Kasungu explaining"

In the past, people who are affected [by HIV] were just staying in darkness without knowing that in future there is peace … they are now staying without worries but before they were unhappy thinking of their status.

The providers indicated that the referral system helped them better understand the full array of services available to clients and how to adequately address client health and livelihood needs. One stakeholder from Kalembo Health Centre in Balaka said:

What makes me happy is that I remember we did a campaign; we found out that people had problems and we didn't know where to take those problems to … When the organizations came together [in the referral system], it was like we have advertised them so that people should know what they do, and if they need a service they now know where to get it.

Health facility staff expressed how they believed referrals reduced the number of ART defaulters and allowed them to find lost clients more easily. One participant from Diamphwe Health Centre in Lilongwe said"

Those who stopped [attending their appointments] some time back, they started now coming because of the advantages of this project, and they are coming to the hospital to disclose themselves … [They say,] “I am your client and stopped coming for a couple of years because of other problems [and] now I am back.”

The health care providers reported being able to trace clients through VSLAs and more easily check in with their service provider colleagues. In Kasungu and Lilongwe, particularly, training community-based referral actors, such as Referral Volunteers and Village Agents, to support the NCST staff in tracking and counseling PLHIV who have been lost to follow-up was seen as helpful by the majority of FGD participants. One from Matapila Health Centre in Lilongwe mentioned monthly coordination and follow-up meetings:

… we conduct meetings with the volunteers once a month when we discuss why "so so" person is not coming … as a result of strategies to reach defaulters, a lot of who were not coming have started coming indeed.

In addition, health facility staff in all FGDs expressed positive value for the time required to participate in the referral system. A stakeholder from Kalembo Health Centre in Balaka explained:

I have seen that this program is not special work for us. We can say it is more like quality control, which can make us have good work, even though when you are working you think you are just wasting time but at the end you see the benefits of what you have done.

### Demand for Provider Services

In Kasungu and Lilongwe, providers thought that demand for economic strengthening (particularly VSLA) and health services increased as a result of their participation in referral networks. Increased client interest in joining VSLAs was cited in all 4 focus groups. Several FGD participants took this a bit further by tying increased demand for and access to economic strengthening support directly to improved outcomes for PLHIV. One referral volunteer responsible for following up with clients to ensure referral completion in Kasungu explained:

It happens that a person is on medication, so because of how people in the village are treating the person, maybe he/she needs a little money to use it for milling, or when the child is sick, or for school fees. But the person fails to borrow or [others] refuse to lend to [the person] thinking, "Is my neighbor going to manage to pay me back with the way she looks?" Now because she has joined the group and she is allowed to borrow that [VSLA social fund] money, she can use that to pay school fees or for milling and has received help for her household.

### Referral Tools

In Balaka, where paper tools and reference guides supplemented the mobile mHealth case management system, LIFT received automated reports documenting referral partner activity. Despite ongoing technical assistance and training, institutionalization of the technology was never fully maximized. This topic came up in each Balaka FGD, with one stakeholder from a community-based organization explaining:

… to follow up a client you have referred to a certain organization, sometimes it happens that you don't have airtime.

However, the project gave all referral providers in Balaka data bundles specifically meant for mobile phone and mHealth app use, which should have allowed them to check referral completion in real time and indicate whether they were currently able to accept new referrals.

Despite ongoing technical assistance and training, referral providers in Balaka were not able to fully maximize use of the mHealth app.

The use of mobile phones to collect data from clients was also an issue in Balaka. In both Balaka FGDs, providers reported that a small minority of clients were afraid of the mobile phones and connected them with Satanism, a belief that took time and energy to dispel during normal operations. Client culture and beliefs must always be considered to prevent project activities from causing alienation or fear.

A few Balaka FGD participants did not seem to understand how to properly apply paper tools to supplement the mHealth app in support of clients. One non-health care provider in Balaka suggested:

Maybe LIFT should have a directory by area. [So we can say to clients], "You come from such and such area, so the organization that can help you is so and so."

In reality, LIFT helped develop and update a service directory in Balaka that was distributed to all referral network members. The network members were encouraged to use monthly meetings to inform one another of critical programmatic changes that would influence the appropriateness of referrals. These examples indicate insufficient understanding of app functionality, the purpose of supplemental paper tools, and the value of regular monthly meetings as fora for information sharing.

In contrast, few suggestions to improve the referral tools used in Kasungu and Lilongwe emerged during the FGDs. The tools used there were simple, consisting of an enrollment form, a referral register, and a paper card that clients could carry to their referral appointment. Tools were entirely paper-based, so they did not require learning to use a mobile phone for referral data entry. Because the referral system linked clients from only clinics (for ART services or food aid) and VSLAs, no robust service directory was needed.

### Referral Level of Effort

The burden of labor on the providers varied widely. Some FGD participants felt they did not have the time to appropriately follow the referral process from initiation to completion, while others found the set-up very user-friendly and simple. Most health care providers reported that they were not overwhelmed by added referral responsibilities, mentioning how they successfully integrated referral messaging into regularly scheduled group ART counseling sessions, and how individual referral sessions were not overly time-consuming. One health care provider from Kasungu District Hospital explained:

With one person, you spend maybe 10 minutes.

### Provider Perceptions of Client Understanding

FGD participants believed that some of their clients misunderstood the referral process. For example, when a provider refers a client to a microfinance organization such as Vision Fund Malawi, the client may eventually be able to obtain a loan through the organization, but this is not guaranteed immediately. A non-health care provider from a community savings organization in Balaka explained:

If we tell them that we are connecting you to food services, they think that they are going to receive food there.

All providers have certain criteria that clients must meet to be eligible for their services, and LIFT worked with referral network members to capture this critical information in tools such as service directories. However, not all service providers referenced these tools effectively. As a result, some clients were frustrated when they realized that they must meet certain eligibility requirements before they could join a VSLA or receive other support, such as food. There is a need to understand, plan for, and balance what facilities counsel clients on during routine ART sessions, such as in this case where direct food aid was beyond the scope of the project.

Some clients did not understand that they had to meet certain requirements before becoming eligible to obtain the referral service.

### Requests Beyond the LIFT Project Scope

On at least one occasion during all FGDs, the participants expressed a desire for material benefits to participating in the referral system, such as lunches and financial support, that are beyond the LIFT scope of work. For example, during an FGD with health care providers in Balaka, a provider from Balaka District Hospital said the following about monthly meetings:

It happens that people who attend [the meetings] are few. … the problem is that in these meetings, most of the times they only have refreshments and maybe transport [allowances] for those from far. We stay at the meeting for hours, but if they can put in a lunch allowance it can encourage people to come.

LIFT purposely limited meetings to discuss operational issues and review referral data to 1 hour only, because half-day meetings are costlier and there was often not enough to discuss to fill a longer agenda.

Many providers felt LIFT's geographic focus was too limited and requested project expansion beyond our scope. For example, one non-health care provider in Balaka explained:

The organizations that are in network do not cover all the areas in Balaka. Sometimes it happened that in the Traditional Authority [area] where the person is coming from, the area doesn't have the [needed] services of an organization, so that was a challenge.

This implies LIFT should expand coverage, but it also relates to use of referral tools mentioned previously, since all stakeholders were given a service directory that described geographic coverage for various programs. Most FGD participants saw the value of participating in referral networks supported by LIFT—for themselves and their clients—but wanted LIFT to work within a catchment area larger than was originally targeted so that more clients could benefit.

## DISCUSSION

The findings from this assessment are useful for integrated, multisectoral development approaches that operate under the hypothesis that beneficiaries receiving a service in one sector will experience improved outcomes in another. In the case of the LIFT project in Malawi, clients received referrals to and from health, economic strengthening, livelihoods, or food security services, regardless of HIV status. We facilitated implementation of 2 types of referral models: (1) a robust model in Balaka district that included more than 20 types of service providers and that used an mHealth data collection and follow-up mechanism, and (2) a simplified model in Kasungu and Lilongwe that connected clients from nutrition support facilities to savings and loan associations using a paper-based referral system. While both referral models successfully linked clients to new services, and all interviewed clients reported positive experience and improvements, the referral models had key differences, discussed below in 4 areas: referral completion, client health outcomes, referral tools, and operational lessons for referral programming.

### Referral Completion

The decision process employed by clients when weighing whether to spend their valuable yet limited resources (time, money, energy, etc.) in order to act on a referral is not always straightforward. While clients need the assistance offered through the referral, they likely face tradeoffs, such as using money for transport to travel to service provider locations versus staying home to tend to their children, business, or crops. It is vital for stakeholders to understand the choices clients confront. If providers refer clients to another service only to find upon arrival that the service is no longer available or that the client is ineligible for the service, the referral system will struggle and mistrust of service providers will build.

Barriers to completing referrals—whether from a health facility to the community or vice versa—are numerous and include food insecurity, transportation costs, income cuts, or lost opportunity costs from missing work, among others. It is beneficial to continuously map these barriers and discuss them with stakeholders in the planning stages of a referral network. In addition to understanding client barriers to referrals, objections may also come from the providers themselves who are new to the idea of multisectoral referrals. For example, some health facility staff may not see the benefit of providing referrals to non-health services, even if the expectation is that client participation in those services may improve health outcomes.

Clients face many barriers to completing referrals including food insecurity, transportation costs, income cuts, and lost opportunity costs from missing work.

For staff managing referrals, it is essential to undertake a detailed analysis of client barriers from the start of fieldwork, ideally before referral tools are developed. In addition, network providers should discuss these barriers at regular referral network meetings. In LIFT's experience, providers will be enthusiastic to begin referrals but may not fully be able to articulate the referral process to clients, including what kind of assistance may (or may not) be available to help overcome their barriers. All providers must know what incentives exist (such as transportation reimbursement or other schemes) that will help clients overcome barriers to referral completion. Further, clients who have completed referrals should be contacted to learn about their experience in the referral system. This can be done by a technical assistance partner such as LIFT, or the providers that make up the referral network can invite clients to participate in monthly meetings to share their stories.

### Client Health Outcomes

The positive health benefits clients and providers attributed to referrals should not be ignored, yet it is important to ensure that perceptions are grounded in reality. A review of facility-level ART unit reports revealed that the clinical default rate remained consistently high (around 15% per quarter) over the study period, indicating that some providers participating in the FGDs were likely overeager to attribute a perceived change in the default rate to the referral system.

In Kasungu and Lilongwe, most referrals to VSLAs originated from health facilities where the referral sensitization process was part of the normal ART care and treatment protocol during clinic days. It is possible that because of the direct and central involvement of clinical staff in this referral work, clients felt more comfortable participating than if being engaged by staff from the community as a PLHIV.

For referral managers, it is important to collect data from multiple sources when assessing client outcomes. This is especially true regarding adherence to ART where social desirability bias can lead clients to indicate they comply with medication dosages and frequencies, but in reality do not do so based on a medical record completed by health facility staff. This kind of outcome data collection must be planned well in advance to ensure stakeholders understand why it is being done, and also to follow ethical review processes and approval by local authorities.

### Referral Tools

Design of integrated, multisectoral referral networks requires careful consideration of the kinds of data that stakeholders will need to collect and how. In Kasungu and Lilongwe, simple paper-based data collection booklets and referral cards were employed; in Balaka, LIFT worked with service providers to design mobile-based survey forms that could be linked as part of a digital case management system. The mobile system, housed on LIFT-provided smartphones, allowed functionality beyond that of paper-based tools, such as real-time data sharing, controlled question trees that limit user error, and incorporation of metrics that "diagnose" client needs and produce referral service recommendations based on client household food security and poverty levels. While this technology was received warmly by all referral network members and government stakeholders, in reality, comprehension of the effort needed in order to actualize the benefits was unbalanced. Even with ongoing technical assistance, repeated training on use of these tools, and incorporation of user feedback into tool revisions on multiple occasions, LIFT struggled to promote proper and sustained mobile-based tool usage by all referral network members in Balaka. In contrast, the Kasungu and Lilongwe paper-based referral tools required very little training for the providers to use.

While the mobile app used to facilitate referrals in Balaka district had more functionality than the paper system in Kasungu and Lilongwe, providers found the app more difficult.

Program managers should make referral tool design (including data collection and analysis) as participatory as possibly with local stakeholders. A pilot or trial phase is helpful because many stakeholders will not fully appreciate how referral logistics can or cannot be changed until they have several weeks or months of experience. It is also essential that managers clarify their role from the outset. LIFT communicated clearly from the beginning that as a technical assistance mechanism, the project's role was to help equip existing service providers with the tools needed to formalize and augment relationships between each other, to promote collaboration and link vulnerable clients more easily and effectively to the services they need most. Throughout the period of engagement, LIFT stressed that the strength of the network would depend on not only the appropriate application of referral tools and processes but also the buy-in of local leaders and the members themselves. FGD responses, while positive in general, demonstrate a lack of comprehensive understanding of responsibilities on the part of some referral providers.

### Operational Lessons for Referral Programming

The results of this qualitative data collection are in line with LIFT observations from routine technical assistance offered during project operations. At all referral network sites, LIFT carried out informal quality improvement actions on a continual basis to build capacity on tool usage and process application. Specific technical assistance was provided to the lead organization in Balaka and health facility referral hubs in Kasungu and Lilongwe to support their coordination roles. Data collected by frontline staff were compiled, analyzed, verified, and shared with all network members, often during regular monthly meetings. These monthly meetings served as fora to discuss what worked and did not work for providers, to share experiences among the members, and to review the most recent data to provide a picture of referral network strength and reach as well as to promote accountability among members.

LIFT considered these capacity building and data sharing activities integral to project plans, but the feedback collected during FGDs may have been different had quality improvement aims and objectives been tied more directly to network performance. In addition, it would have been helpful for LIFT to create data management dashboards and analysis tools as well as train local stakeholders to use them earlier on—these tools were eventually developed but only toward the end of LIFT's engagement in country. Each of these items could have helped promote accountability. LIFT sought to help stakeholders collectively develop action plans to set and achieve goals, but more could have been done in Malawi. LIFT is now incorporating quality improvement concepts within all current referral work in other countries.

LIFT always sought to be clear with stakeholders on the project's role and purpose. Repetition and transparency were key to effectively communicate the message, yet LIFT still struggled to ensure uniform comprehension of the project's responsibilities and limits to the support provided. During this assessment, providers in the FGDs brought up several instances where the scope of LIFT was not clearly understood.

Staff turnover is a reality in any line of work, and although it was not explicitly mentioned as a challenge in the findings of this assessment, LIFT's experience demonstrates the importance of adequately planning for these transitions. Initially in Balaka, LIFT left the decision up to the network members regarding whom from their offices should serve as focal persons for referral work. In many cases, the person chosen was not well-positioned to assume added referral responsibilities, nor did they have regular interaction with prospective referral clients on a daily basis. Several focal persons held positions that required them to travel frequently and/or be present in town offices where client traffic was minimal. As implementation progressed, LIFT worked with the network members to train additional staff who were often better placed to manage direct client interaction. This lesson highlights the fine balance between referral network autonomy—allowing members the freedom to make their own choices—and LIFT assertively guiding stakeholders toward specific choices from the beginning.

In Balaka, LIFT sought to use an mHealth app that would be easy to adapt for referral purposes, provide value for money, and be user-friendly. While LIFT did seek out and incorporate stakeholder feedback from the beginning, this type of iterative process may not have been the most effective toward promoting local ownership and accountability. Alternatively, LIFT could have proposed other tool choices for referral stakeholders to assess and then worked at a slower pace to test and adapt until all stakeholders were comfortable and committed. The usage of technology necessitated constant follow-up and troubleshooting. As a technical assistance partner, LIFT had hoped to transfer capacity to local partners, yet the institutionalization of the mHealth tools proved to be a considerable challenge.

Related to this, LIFT's ongoing technical assistance visits revealed how network members did not always honor referrals made to their organization. During training and the early months of referral work, LIFT helped the members think about their capacity to serve new clients. If they could not responsibly serve new clients, strategies to temporarily divert referrals were discussed—for example, attending monthly meetings and explaining this situation to the other network members.

This highlights the local ownership and management imperative of referral system development; network members themselves are responsible for updating tools and informing one another if they are unable to provide services to potential referral clients.

### Limitations

This assessment has several limitations, yet provides a good deal of summative evaluation information for future multisectoral referral work, most notably how to engage stakeholders in tool development, referral operations, and understanding how referrals affect their clients. In order to make a claim of causation concerning referrals (i.e., that participation in a referral directly improved clients' household resilience), more complex study designs are required. Other limitations include:

- Many clients who were registered and referred using materials provided by the LIFT project were encountered only once prior to being interviewed. LIFT did not track in-stances where stakeholders may have attempted to follow up with clients on any issues related to referrals.
- The majority of the clients interviewed were referred at some point within the year prior; depending on the referral service received, the time elapsed between the referral and the interview could have limited client perception of utility or benefit. For example, if clients were referred to a VSLA group within 6 months of the interview, they may not have had the opportunity to fully pass through a savings cycle to realize a discernible value.
- The data are not expected to be generalizable beyond the context of referrals in Balaka, Kasungu, and Lilongwe. They are being collected to help guide programming in the area, to produce recommendations for tools to be used, and ultimately to provide guidance that is useful for referral systems in Malawi.
- No audit or review of network provider service registers was conducted to verify statements made by FGD participants.

In the future, LIFT would recommend more statistically rigorous methods to explore the linkages between adherence and retention for PLHIV and participation in other activities and services. For example, a non-intervention community could be selected to serve as a counterfactual. LIFT project staff did complement this qualitative work with a longitudinal medical record check to determine if referral clients had improved adherence to and retention in care and treatment programs, which will be reported separately once endline data are collected and analyzed.

## CONCLUSION

Integrated referral systems with formal processes and standardized tools that allow network providers to effectively track and monitor client participation and referral completion can improve the ability of service providers to meet more fully the holistic demands of their clients. Clients—both those with and without HIV infection—referred by LIFT-supported stakeholders in 3 districts of Malawi perceived their referral as positively influencing their own and/or their family's health and wellness, specifically when it came to adherence to medications and ability to save money. The network providers, encompassing health facilities and community economic strengthening, livelihoods, and food security services, not only substantiated these self-reported client claims but also indicated that their active participating in the referral network has helped them promote their own work while broadening their client reach.

An important operational lesson for other multisectoral referral efforts is to limit the number of referral services available in the network. The Balaka referral network had more than 20 services, which made it difficult for network providers to keep track of important updates in service availability, program start and end dates, points of contact, etc., even with a printed service directory that contained that information. The Kasungu and Lilongwe referral networks technically had 3 services (nutrition support, food aid, and savings and loan support), but only 2 service delivery points since food aid (when available) was disbursed at the NCST facility. This more streamlined system was easier to administer and explain to clients. Further, referral tools should be as simple as possible to ease uptake and transfer of knowledge to staff supporting referrals. While the mHealth app used in Balaka was robust, it required significant training and a steep learning curve, while the paper forms used in Kasungu and Lilongwe (with the added support of referral volunteers) were easier for the providers to learn and use, even though the data entry and aggregation took more work.

## Notes

**Peer Reviewed**
